# Antiestrogen- and tamoxifen-induced effects on calcium-activated chloride currents in epithelial cells carrying the ∆F508-CFTR point mutation

**DOI:** 10.1186/s12931-018-0901-1

**Published:** 2018-10-05

**Authors:** Roberto Imberti, Maria Lisa Garavaglia, Ivan Verduci, Gaetano Cannavale, Giorgio Balduzzi, Sara Papetti, Michele Mazzanti

**Affiliations:** 1 Phase I Clinical Trials Unit and Experimental Therapy, Fondazione IRCCS Policlinico San Matteo, 27100 Pavia, Italy; 2 Department of Biosciences, Laboratory of Cellular and Molecular Physiology, University of Milano, via Celoria 26, I-20133 Milan, Italy; 3 GB Pharma S.r.l., via Ferreri 11, 27100 Pavia, Italy

**Keywords:** Chloride current, CFTR mutation, CaCC, Ion channels, Tamoxifen, 17β-estradiol

## Abstract

**Background:**

Although pharmacological treatment has increased the average life expectancy of patients with cystic fibrosis, the median survival of females is shorter than that of males. In vitro and in vivo studies have shown that estrogens play a relevant role in the disease progression.

The aim of this study was to investigate the effects of 17β-estradiol and tamoxifen citrate (TMX) on calcium-activated chloride channel (CaCC) currents in human bronchial epithelial cells carrying the ΔPhe508-CFTR mutation both in homozygosis and in heterozygosis.

**Methods:**

Perforated patch clamp experiments were performed on single cells of the immortalized cell lines CFBE and IB3–1. Gramicidin (10 or 20 μM) was added to the electrode solution to reach the whole cell configuration. The electrical stimulation protocol consisted of square voltages ranging from − 80 to + 80 mV, in steps of 20 mV and with a duration of 800 msec.

**Results:**

The presence of 17β-estradiol significantly reduced the CaCC currents, both in basal conditions and in the presence of ATP (100 μM). The addition of TMX (10 μM) completely restored the currents abolished by 17β-estradiol, in basal conditions and after stimulation with ATP in both CFBE and IB3–1 cells. TMX had a strong, direct action on membrane current density, which significantly increased more than 4-fold in both cases. The membrane current stimulation produced by TMX was further enhanced by the addition of ATP. CFBE cells incubated for 24 h with 3 μM VX-809 (a CFTR corrector) and then acutely stimulated with VX-770 (a CFTR potentiator) in the presence of forskolin, showed an increase of chloride currents which were abolished by Inh-172. The chloride current density induced by TMX + ATP was, on average, greater than that obtained with VX-809 + VX-770 + forskolin. The currents elicited by TMX + ATP were abolished by the addition of NPPB, a CaCC inhibitor. The combined administration of TMX/ATP and VXs/FSK had an additional effect on chloride currents.

**Conclusions:**

Our results show that TMX restores CaCC currents inhibited by 17ß-estradiol and directly activates the transmembrane chloride currents potentiated by ATP, an effect which is mutation independent. The combined effect of TMX with current used treatments for cystic fibrosis could be of benefit to patients.

## Background

Cystic fibrosis (CF) is the most common fatal genetic disease in the white population. It is caused by mutations in a gene encoding the Cystic Fibrosis Transmembrane Conductance Regulator (CFTR), a protein that functions mainly as a chloride channel. A mutation of the *CFTR* gene usually produces abnormal proteins that do not transport chloride ions and water properly, or are not transported to the apical membrane [1–3]. More than 2000 genetic CFTR variants are known, the most frequent being the F508del. Most mutations of the *CFTR* gene are missense alterations, but frameshifts, splicing, nonsense mutations, and in-frame deletions and insertions have been described. About 15% of the genetic variants that have been identified are not associated with the disease [3] . The CFTR channel defect is mainly in chloride and bicarbonate transport. Interactions of CFTR and other ion channels, particularly the epithelial sodium channel, and interactions of CFTR with cellular pathways related to inflammation (inflammasome) might be important in the pathophysiology of CF [4]. The importance of understanding the pathophysiology of this disease in the first few years of life has been underscored by recent studies showing that, by the age of 3 years, almost a third of children with CF have computed tomographic evidence of mucus obstruction, bronchiectasis, and inflammation driven by neutrophils, neutrophil elastase, and recurrent episodes of infection [4, 5]. The primary hypothesis to explain these clinical features is that impaired mucociliary clearance caused by abnormal hydration of airway surface liquid is the key underlying defect [4, 5]. In newborn pigs with CF it has been observed that mucus fails to detach from submucosal gland ducts and accumulates in pulmonary airways, thus hindering mucociliary transport, an abnormality which, at the origin of the disease, is not dependent on infection or inflammation [6]. With progression of the disease, advancing infection and bronchiectasis further disrupt mucociliary transport, which, in turn, impairs bacterial clearance and promotes resistance to antibacterial defenses [4].

Although CF is not sex-linked, females with this disease experience a more rapid decline in lung function, have more pulmonary exacerbations and have a shorter life span compared with males with CF [7, 8]. Several lines of evidence indicate that the female sex hormone estrogen plays a relevant role. In vitro studies have shown that estrogen receptors ERα and ERβ are expressed in normal lung tissue [9, 10] and that ERα are expressed in cell cultures from non-CF and CF patients, at similar levels in males and females [10]. Choi and colleagues [11] have shown that 17β-estradiol, by interacting with ERα, up-regulates *MUC5B* gene expression and increases the production of MUC5B, one of the major mucins in the human airway submucosal glands [12], thus favoring mucus plugging. Pretreatment with the estrogen receptor antagonist ICI182,780 suppressed 17β-estradiol-induced MUC5B expression [11]. Inflammation is another important mechanism of lung injury and lung function decline in CF patients. A recent investigation demonstrated that exogenous administration of 17β-estradiol to male CF mice increases the severity of *P. aeruginosa* pneumonia by increasing inflammation and the production of mucin and inflammatory cytokines [13]. Furthermore, circulating levels of estradiol correlated with infective exacerbations among menstruating women with CF, and the exacerbation rate per year was significantly lower in women receiving oral contraceptives than in CF females with a regular or irregular menstrual cycle [14]. Moreover, exposure of *P. aeruginosa* to estradiol resulted in an increase of alginate, the predominant polysaccharide produced by *P. aeruginosa* and implicated in its pathogenicity, and enhanced the conversion from the non-mucoid to the mucoid morphology [14].

Another recent investigation showed that 17ß-estradiol, by reducing the ATP-induced increase in intracellular Ca^2+^, inhibits the activity of calcium-activated chloride channel (CaCC) [10]. Treatment with tamoxifen citrate (TMX) restored the ATP-induced increase in intracellular Ca^2+^ despite the presence of 17ß-estradiol, thus increasing airway surface liquid production. TMX was more potent than the pure ER antagonist ICI182780. However, direct measurements of the effects of TMX on CaCC currents are not available.

The aim of this study was to determine the effects of 17β-estradiol and TMX on CaCC currents in human bronchial epithelial cells carrying the ΔPhe508-*CFTR* mutation both in homozygosis and in heterozygosis. The increase of chloride membrane current promoted by the application of TMX could be an additional support to induce transepithelial water flux. Ultimately, the goal was to investigate the potential role of TMX as a co-adjuvant to existing pharmacological therapies for CF.

## Methods

### Cell cultures

**Fig. 1:**
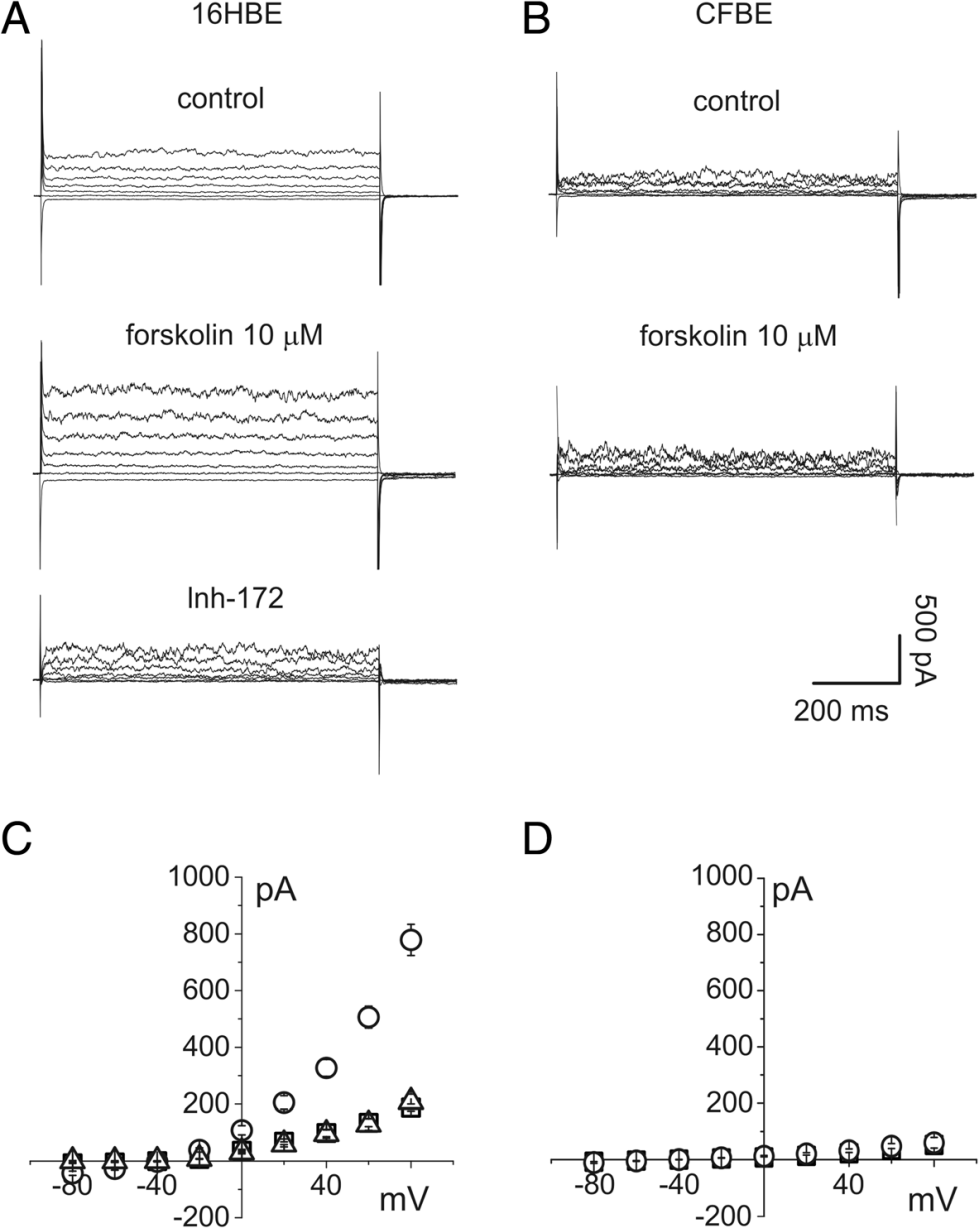
Total membrane current of 16HBE and CFBE epithelial cells elicited by ±80 mV voltage in 20 mV steps. Holding potential − 40 mV. **a** whole cell current recordings from single 16HBE cells in the control condition (top), after stimulation with 10 μM forskolin (middle) and after perfusion with the CFTR ion channel inhibitor Inh-172 (bottom). **b** whole cell current recordings from CFBE single cells in the control condition (top), after stimulation with 10 μM forskolin (bottom). **c** current/voltage relationships of 16HBE total membrane currents in the control condition (squares), after addition of 10 μM forskolin (circles; *n* = 6) and in the presence of Inh-172 (triangles; *n* = 3). **d** current/voltage relationships of CFBE total membrane currents in the control condition (squares *n* = 5) and after addition of 10 μM forskolin (circles; *n* = 7)

Electrophysiological measurements were performed on single cells of the immortalized cell lines CFBE and IB3–1, created from primary cultures of human bronchial epithelial cells carrying the F508del/F508del [15] and F508del/W1282X [16] mutation, respectively. Cells were maintained in an incubator at 37 °C in 5% CO_2_, and seeded in 35 mm Petri dishes the day before the experiment. We also used 16HBE epithelial cells as a control in the experiment whose results are shown in Fig. 1.

### Electrophysiology

Perforated patch clamp experiments were performed according to a standard technique. Gramicidin (10 or 20 μM) was added to the electrode solution to reach the whole cell configuration. The temperature at the recording site was monitored and its average value, determined from ten different measurements on different days, was 28 °C ± 2 °C. Cells were bathed with a solution composed of 150 mM NMDG-Cl, 1 mM MgCl_2_, 2 mM CaCl_2_, 10mMHEPES, 10 mM glucose, and 30 mM mannitol or raffinose, pH 7.4. The extracellular solution was kept hypertonic (337 mOsm) in order to inhibit currents generated by cell swelling. The different compounds tested in this study were added to the external solution at the concentration and at the time of incubation reported on each plot. The pH of the external solution was tested after addition of every molecule used in our experiments. A fast perfusion system was used to deliver the different compounds acutely or to ensure that the cells were constantly in the presence of the drug used in the incubation phase. The patch electrode was pulled with a Sutter P97 horizontal puller to a tip diameter of 1–2 μm and a resistance of 5–7 MΩ and filled with 145 mM KCl, 1 mM MgCl_2_, 0.5 mM CaCl_2_, and 10 mM HEPES, pH 7.25 to which gramicidin was added just before the experiment. The electrical stimulation protocol consisted of square voltages from − 80 to + 80 mV, with 20 mV steps and a duration of 800 msec. Cell medium was discarded from the Petri dish and replaced with the extracellular solution at the beginning of the experiment. Continuous extracellular solution was perfused by means of an automated perfusion system (RSC-200, Bio-Logic Science Instruments, FR). After getting complete electrical access to the cell, currents were recorded every minute up to register at least three stable traces. Different test solutions were perfused accordingly with the same protocol. Solution change around the cell under observation occurred in less than a second.

3,17β-dihydroxy-1,3,5(10)-estratriene(17β-estradiol), (Z)-1-(p-dimethylaminoethoxyphenyl)-1,2-diphenyl-1- butene(tamoxifen citrate, TMX), 7β-acetoxy-8,13-epoxy-1α,6β,9α-trihydroxylabd-14-en-11-one (forskolin, FSK), 3-[(3-trifluoromethyl)phenyl]-5-[(4-c'arboxyphenyl)methylene]-2-thioxo-4-thiazolidinone (Inh-172) and 5-nitro-2-(3-phenylpropylamino)benzoic acid (NPPB) were purchased from SIGMA, Italy [17]. Ivacaftor (VX-770) and lumacaftor (VX-809) (Selleckchem) were purchased from Absourche Diagnostics GmbH, Munchen, Germany. Ivacaftor (VX-770) and lumacaftor (VX-809), recently licensed for the treatment of some CFTR mutations, are able to increase CFTR protein in the cell membrane and to enhance chloride flux [18]. In our study we tested the effects of the association ivacaftor/lumacaftor on chloride currents to compare quantitatively the effects of TMX on chloride currents, and to evaluate, in our setting, a possible synergism between the association Ivacaftor/lumacaftor and TMX.

We considered only experiments with a membrane resistance between 1 and 1.2 GΩ and an access resistance of 40–60 MΩ to ensure a correct current recording. Current amplitude was calculated as an average of three consecutive single current traces at the same test voltage considering only the last 100 msec. Currents are expressed in current/voltage plots as absolute current value (Fig. 1) or as a density using the cell capacitance as a measure of cell surface. We used an Axopatch 200B for current recording and Pclamp9 to collect the experimental current traces. Clampfit 9 was used for analysis of the currents. ORIGIN 9 was used to process the data and an unpaired t-test was applied to calculate the significance of results, with values of *p* < 0.05 considered statistically significant. Quantitative data were collected from experiments performed in triplicate or quadruplicate and expressed as mean ± s.e.m., except for time-lapse experiments (mean ± s.d.). Current density/voltage relationships were analyzed as following: for each condition, we plotted every experiment (the number of trials is reported in the figure legends) and extrapolated the linear regression for the curve (all statistically significant, with R2 ≥ 0.9). For analyses of two conditions, we used a two-sample t-test; otherwise we performed a two-way ANOVA on the slopes of every experimental group. In the box chart plots, the solid lines within the boxes represent the median values. The square within the boxes is the average, and the boxes show the 25th and 75th percentile range of the measured elements. The maximum and minimum values are depicted as horizontal bars, and the symbols to the right of the box represent the numbers of trials.

## Results

Our experiments were based on the hypothesis that stimulation of CaCC currents could restore the permeability to chloride ions that is downregulated by the malfunctioning of the CFTR ion channel. Homozygosity for the 508 mutation in CFBE cells reduces the chloride flux through the plasma membrane almost to zero, thereby preventing the movement of water. Comparing control 16HBE epithelial cells with the CFBE clone (see methods), the absence of the cAMP-activated chloride permeability in the cells carrying the F508^−/−^ point mutation is evident. Perforated patch current recordings obtained in both types of cells are shown in Fig. 1. The effect of perfusion of 10 μM FSK, causing a cytoplasmic increase of cAMP, is evident in 16HBE but not in CFBE cells. In addition, the increase of membrane current in 16HBE cells was totally inhibited by 10 μM Inh-172, a specific CFTR channel blocker. The experiment on CFBE cells, whose results are shown in Fig. 1, demonstrates the absence of CFTR channel activity in the mutated clone [17].

**Fig. 2:**
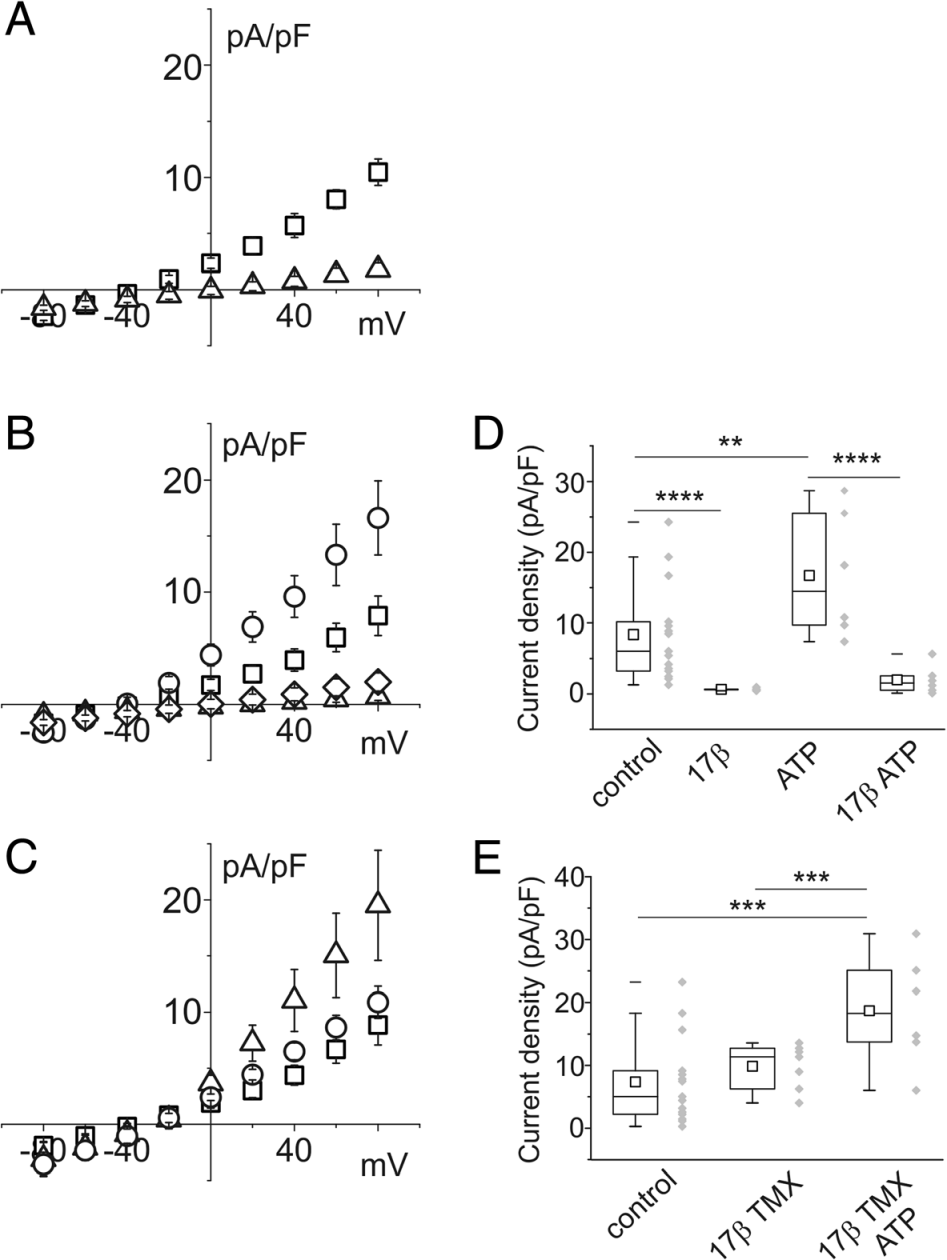
Effect of 17β-estradiol, ATP and tamoxifen (TMX) on CFBE single cell membrane current density. **a** current/voltage relationship of control cells (squares; *n* = 15) and in the presence of the chloride channel blocker NPPB 300 μM (triangles; *n* = 4). **b** current density versus membrane voltages in control cells (squares; n = 15) in the presence of 10 nM 17β-estradiol (triangles; n = 5) or 100 μM ATP (circles; *n* = 6). ATP (100 μM) was not able to increase current density once in combination with 10 nM 17β-estradiol (diamonds; n = 6). **c** current/voltage relationship of control cells (squares; n = 15), after perfusion with 10 μM TMX (circles; n = 7). The addition of 100 μM ATP induced a marked increase of current density (triangles; n = 6). **d** and **e** current density box chart plots at + 80 mV membrane voltage related to the experiments reported in B and C

Perforated patch experiments performed on CFBE cells in control conditions showed a current inversion at − 47.8 ± 6.5 mV, consistent with limited chloride permeability. The basic current of CFBE cells was inhibited by 300 μM NPPB (Fig. 2a). To assess the inhibitory action of the estrogen hormone, CFBE cells were exposed to 10 nM 17β-estradiol for 30 min. The presence of 17β-estradiol significantly reduced the chloride currents, both in basal conditions and in the presence of ATP (100 μM). Activation of the purinergic receptor P2Y_2_ has the effect of CaCC agonism (Fig. 2b and d). However, stimulation with 10 μM TMX induces an increase of intracellular calcium [10]. Accordingly, TMX completely restored the currents abolished by 17β-estradiol, both in basal conditions and after stimulation with ATP (Fig. 2c and e).

**Fig. 3:**
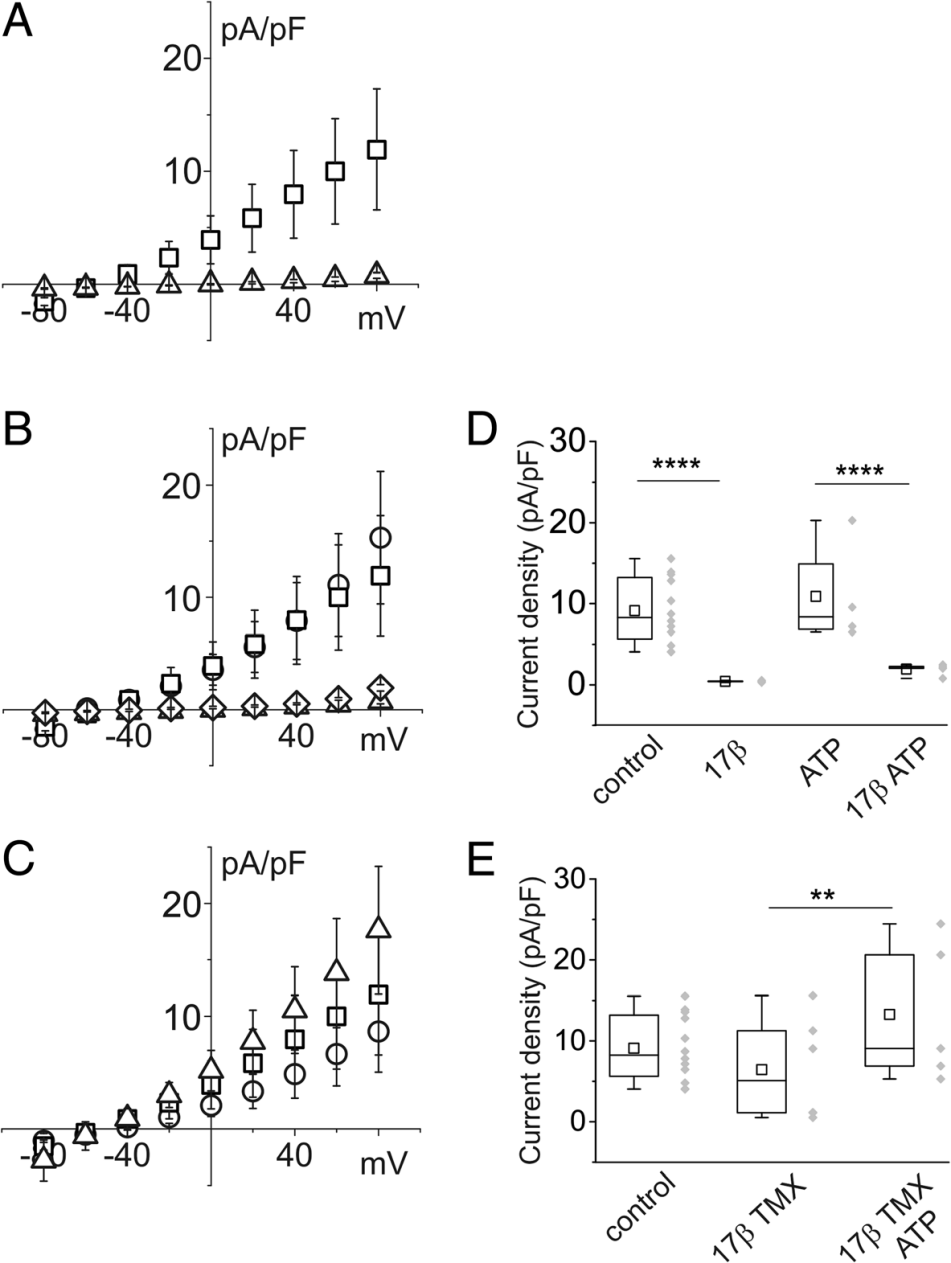
Effect of 17β-estradiol, ATP and tamoxifen on IB3–1 single cell membrane current density. **a** current/voltage relationship of control cells (squares; *n* = 12) and cells in the presence of 300 μM NPPB, a chloride channel blocker (triangles; *n* = 6). **b** current density versus membrane voltages in control cells (squares; n = 12), in the presence of 10 nM 17β-estradiol (triangles; n = 5) or in the presence of 100 μM ATP (circles; n = 4). ATP was not able to increase current density once in combination with 10 nM 17β-estradiol (diamonds; n = 5). **c** current/voltage relationship of control cells (squares; n = 12), after perfusion with 10 μM TMX (circles; n = 6) in the presence of 10 nM 17β-estradiol. The addition of 100 μM ATP induced a marked increase in current density (triangles; n = 5). **d** and **e** current density box chart plots at + 80 mV membrane voltage related to the experiments reported in B and C

The same experimental paradigm was applied to single IB3–1 cells: the findings are illustrated in Fig. 3. Cells with 508 mutation in heterozygosis showed a current reversal potential of − 57.8 ± 7.1 mV, consistent with greater permeability to chloride than that of CFBE cells. Figure 3a shows the effect of 300 μM NPPB on the basic membrane current of IB3–1 cells. IB3–1 cells show a similar behavior to that of CFBE cells after stimulation with 17β-estradiol and ATP (Fig. 3b and d) and in the presence of TMX and ATP simultaneously with estrogen stimulation.

Collectively, these findings indicate that the basic membrane currents recorded in CFBE and IB3–1 cells is mainly carried by chloride through the CaCC.

**Fig. 4:**
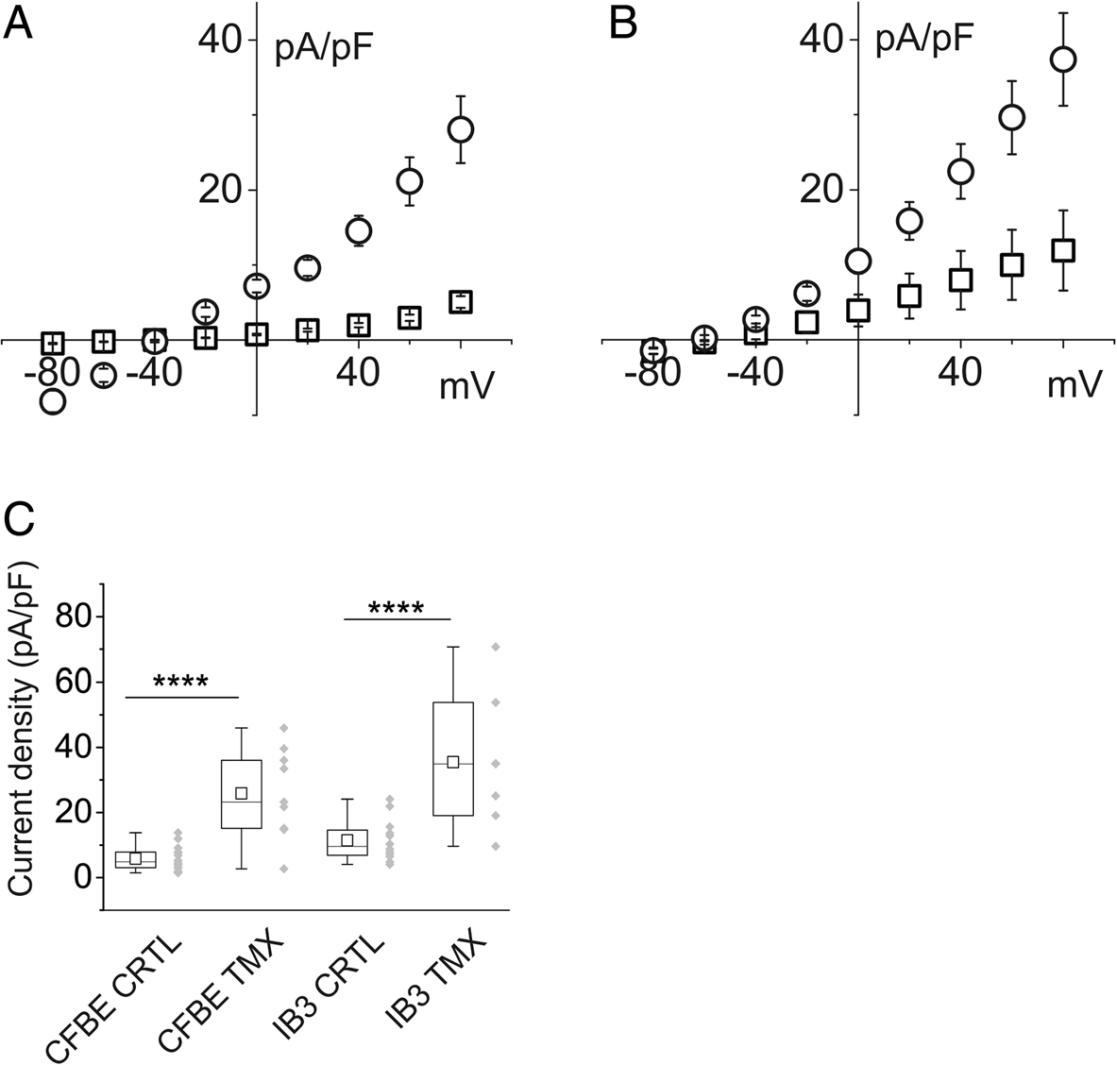
Effect of TMX on current density of single CFBE and IB3–1 epithelial cells. **a** example of current/voltage relationship of CFBE cells in control conditions (squares) and in the presence of 10 μM TMX (circles). **b** example of current/voltage relationship of IB3–1 cells in control conditions (squares) and in the presence of 10 μM TMX (circles). **c** box chart plot of the membrane current density at + 80 mV for CFBE cells in the control condition (*n* = 18) and in the presence of TMX (*n* = 9). The right part of the plot shows average current density for IB3–1 cells in control conditions (*n* = 12) and after exposure to TMX (n = 6)

TMX had a direct effect on membrane ionic permeability of CFBE and IB3–1 cells. Figure 4 shows the results of parallel studies of CFBE and IB3–1 cells stimulated by TMX in perforated patch experiments. Figure 4a and b depict current/voltage relationships for CFBE and IB3–1 cells, respectively, in control conditions and after stimulation with 10 μM TMX.

**Fig. 5:**
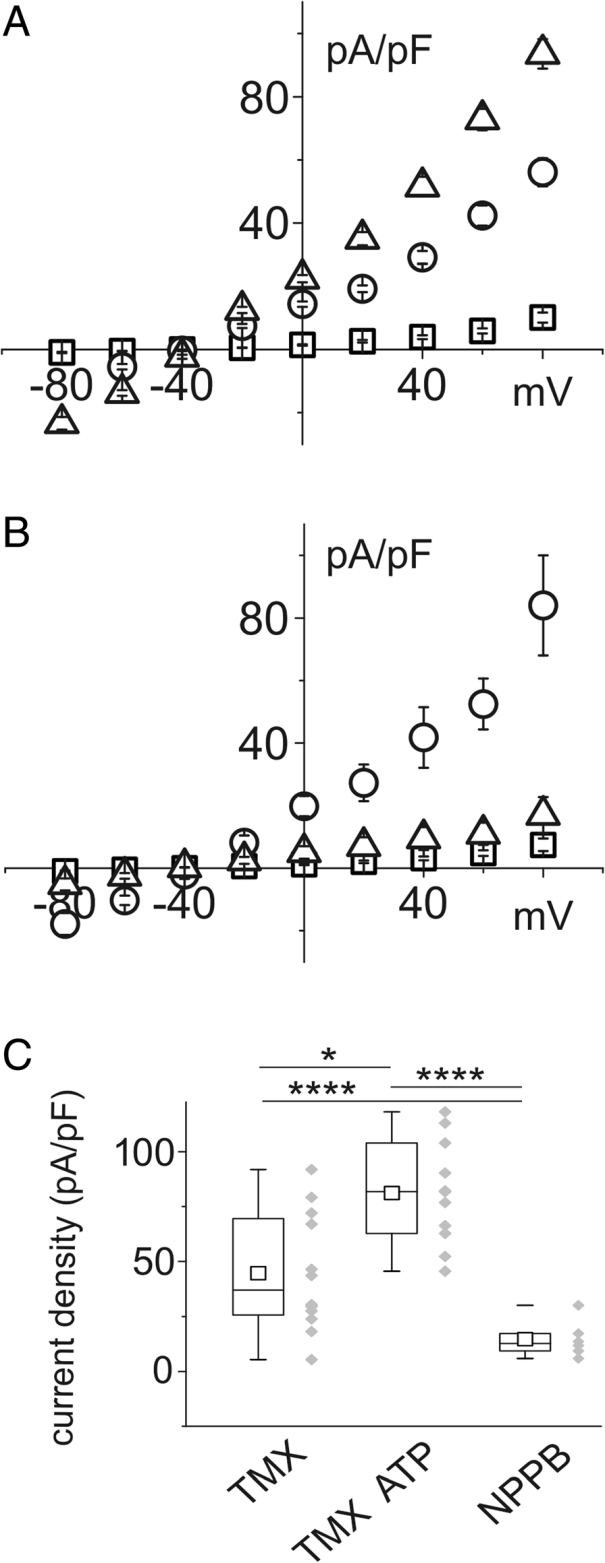
Effect of TMX and ATP on current density in CFBE cells. **a** example of current/voltage relationship of cells in control conditions (squares), in the presence of 10 μM TMX (circles) and after addition of 100 μM ATP (triangles). **b** example of current/voltage relationship of cells in control conditions (squares), and in the presence of 10 μM TMX + 100 μM ATP (circles). Membrane current stimulated by TMX + ATP was almost totally inhibited by 300 μM NPPB (triangles). **c** plot of membrane current density expressed as a percentage of increase at + 80 mV relative to the value of the current in control conditions (*n* = 15; *p* ≥ 0.001). TMX in combination with ATP (*n* = 10) produced a significant increment compared with TMX stimulation alone (*n* = 11; *p* ≥ 0.05). Addition of NPPB completely inhibited the current (n = 6)

The membrane current stimulation produced by TMX (Fig. 5a, circles) can be further enhanced by the addition of 100 μM ATP (Fig. 5a, triangles). Acute application of NPPB totally inhibits the whole cell current stimulated by TMX and ATP (Fig. 5b), confirming that the final targets of TMX and ATP are CaCC.

**Fig. 6:**
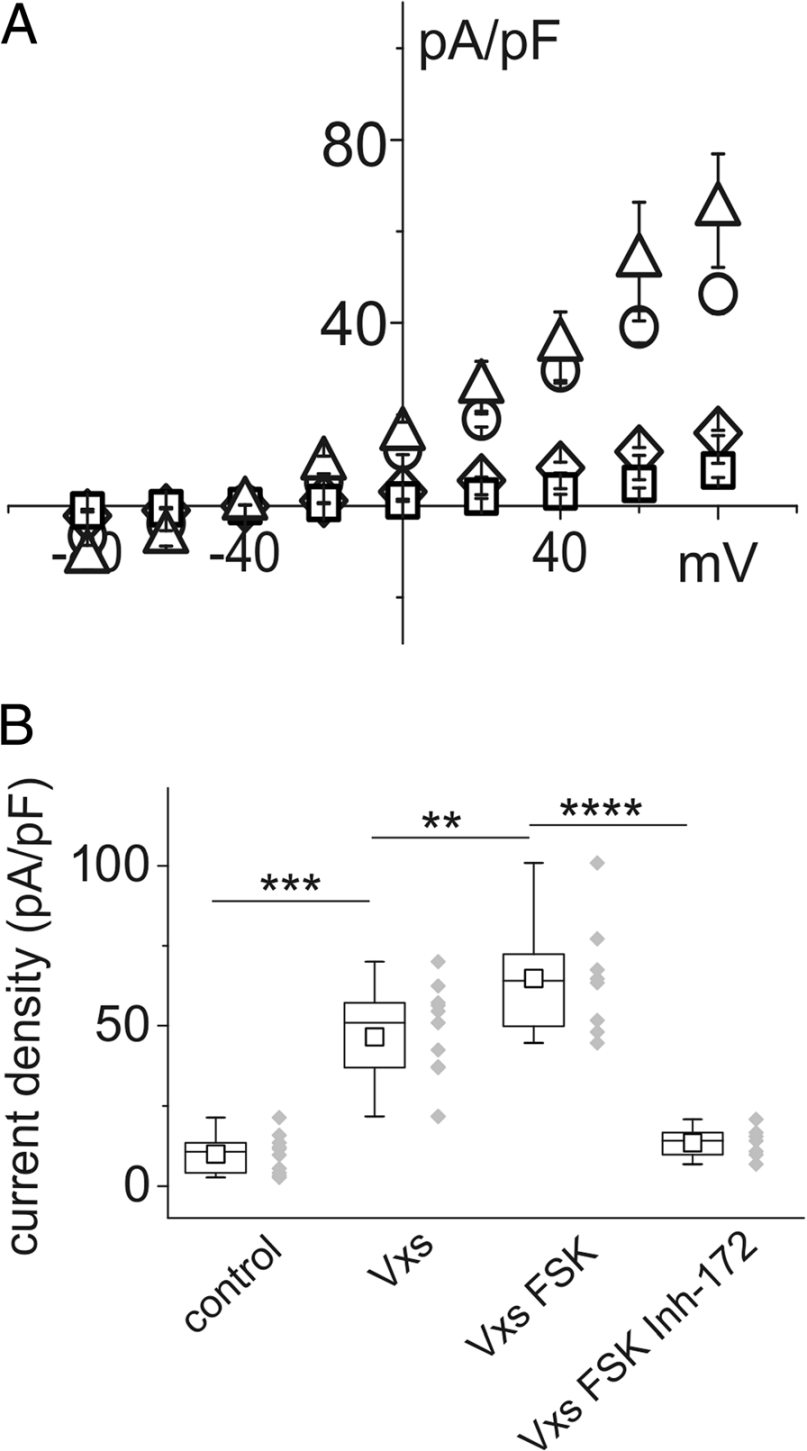
Effect of ivacaftor (VX-770) and lumacaftor (VX-809) on current density of CFBE cells. **a** example of current/voltage relationship of cells in control conditions (squares), in the presence of 1 μM VX-770 and 3 μM VX-809 (circles) and after addition of 10 μM forskolin (triangles). Addition of the CFTR ion channel blocker Inh-172 reduced the transmembrane current almost to that occurring in control conditions (diamonds). **b** chart plot of membrane current density at + 80 mV relative to the value of the current in control conditions (n = 11), stimulated by VX-770 and VX-809 (*n* = 8) and after the addition of forskolin (n = 8) compared to current inhibitor Inh-172 (*n* = 4; p ≥ 0.001)

The in vitro efficacy of TMX on membrane chloride ionic currents in CFBE cells was compared with that of two recently discovered CFTR permeability enhancers. Ivacaftor (VX-770) and lumacaftor (VX-809) are two approved drugs (Orkambi^®^, Vertex Pharmaceuticals) used in combination for the treatment of CF in patients with F508-del mutation in homozygosis. Using an Ussing chamber, Van Goor and colleagues showed that the two drugs increased the CFTR current in experiments performed on a cell monolayer. The procedure enabled the transepithelial current to be recorded in the voltage-clamp mode [18]. In our experiments, we monitored the change of chloride current density on single CFBE cells incubated for 24 h with 3 μM VX-809. Figure 6a shows a current/voltage plot on CFBE cells treated with VX-809 and acutely stimulated with 1 μM VX-770 immediately prior to electrophysiological measurements. At the end of each experiment we also added 10 μM FSK to the external solution. Membrane current amplitude was enhanced upon VX-809/VX-770 exposure; the subsequent application of FSK resulted in a trend towards an increase, although this was not statistically significant. The CFTR blocking agent Inh-172 completely inhibited the membrane current (Fig. 6a and b).

**Fig. 7:**
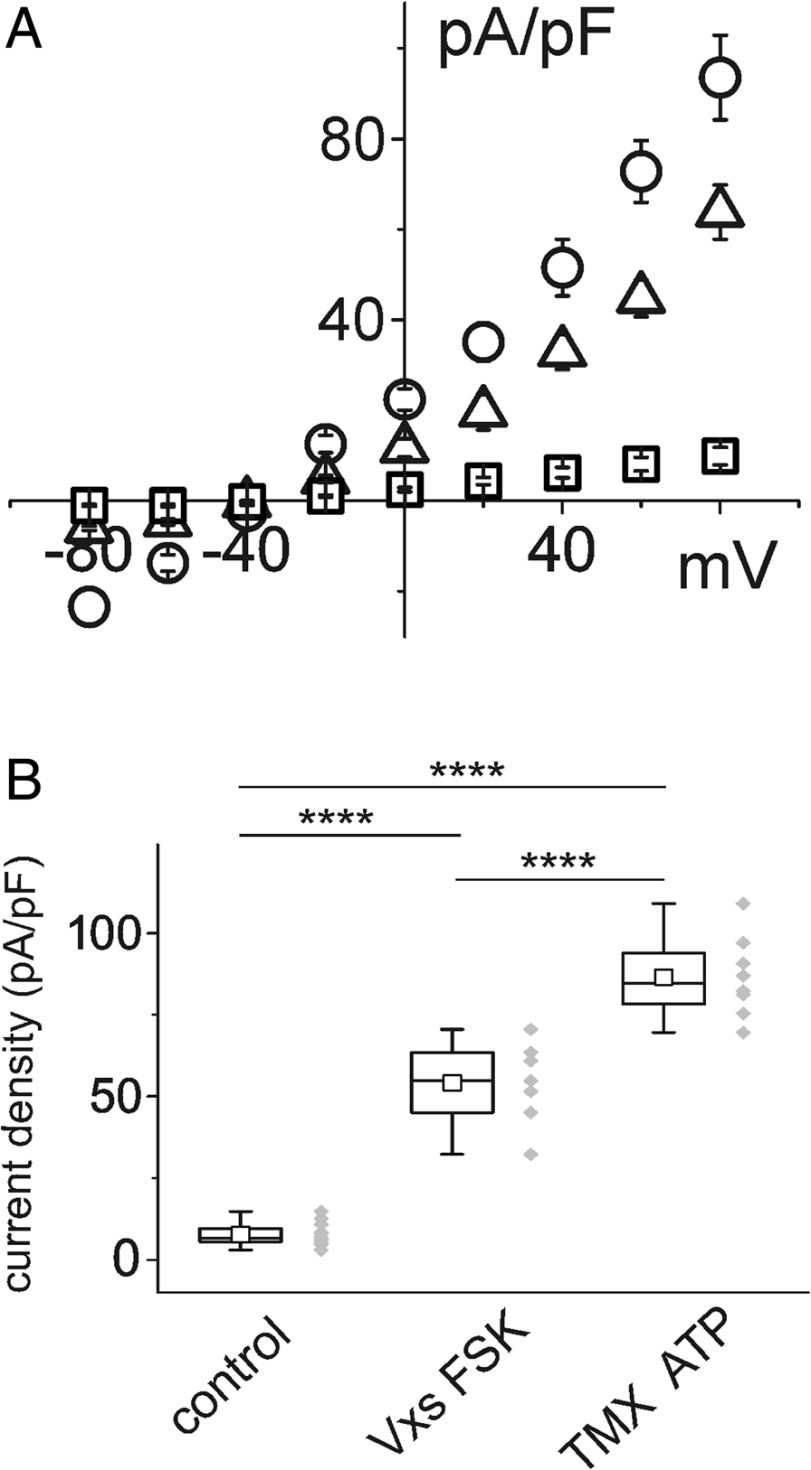
Comparison of the effect of VX-770/VX-809/FSK and TMX/ATP on CFBE cell current density. A: example of current/voltage relationships of cells in control conditions (squares), in the presence of VXs/FSK triangles) and in those treated with TMX/ATP (circles). B: The combination of TMX + ATP (n = 8) showed greater efficiency than VXs/FSK at increasing chloride membrane currents (*n* = 7; *p* < 0.001). Both treatments elicited highly significant increases in current density compared to the density in control conditions (n = 12)

To compare the effects of TMX/ATP with those of VX-809/VX-770/FSK, average current/voltage relationships for both the experimental conditions were plotted (Fig. 7a) and compared with the membrane current in basal conditions. Both curves show a significant increase of the current density. At the 80 mV voltage step, TMX/ATP caused a mean increase in membrane permeability of 29% compared to that produced by VX-809/VX-770/FSK.

**Fig. 8:**
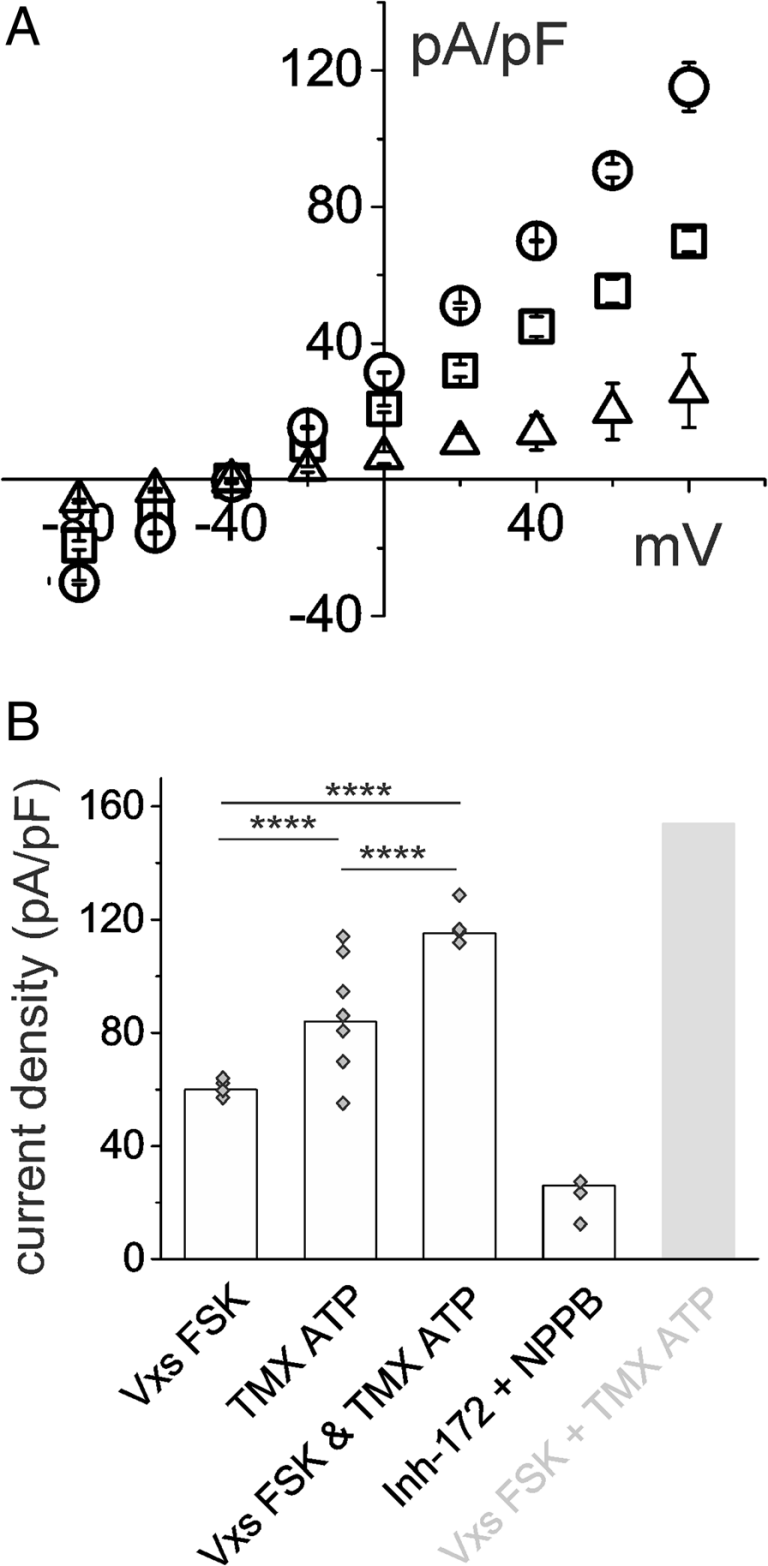
Synergic action of VXs/FSK and TMX/ATP on increasing membrane chloride current in CFBE cells. **a** example of the current/voltage relationship showing current density data from CBE cells after incubation with VXs/FSK (squares) and subsequent addition of TMX/ATP (circles) compared with control condition (triangles). **b** the histogram with the single experiments (grey diamonds) compares current density at + 80 mV membrane potential for CFBE cells stimulated with VXs/FSK (n = 4), TMX/ATP (n = 7) or the combination of both compounds (n = 4). The ion channel blockers Inh-172 and NPPB drastically reduced the membrane current (*n* = 3). The values of the light gray column on the right of the histogram were obtained by mathematical addition of the average current densities of cells stimulated separately with VXs/FSK and TMX/ATP

The combined effects of TMX/ATP and VX-809/VX-770/FSK were seen to be additive. CFBE cells were exposed to VXs/FSK and, after a steady current had been reached, to TMX/ATP. The current/voltage relationship shown in Fig. 8a depicts the membrane current elicited at the different voltages in the presence of VXs/FSK (squares), followed by addition of TMX/ATP (circles) and, finally, perfusion with CFTR and CaCC blockers (triangles). The histogram in Fig. 8b illustrates the different current density values at a membrane voltage of + 80 mV. The average TMX/ATP current value has been imported from Fig. 7. The light gray column on the right of Fig. 8b presents the plain sums of VXs/FSK plus TMX/ATP single averages.

## Discussion

**Fig. 9:**
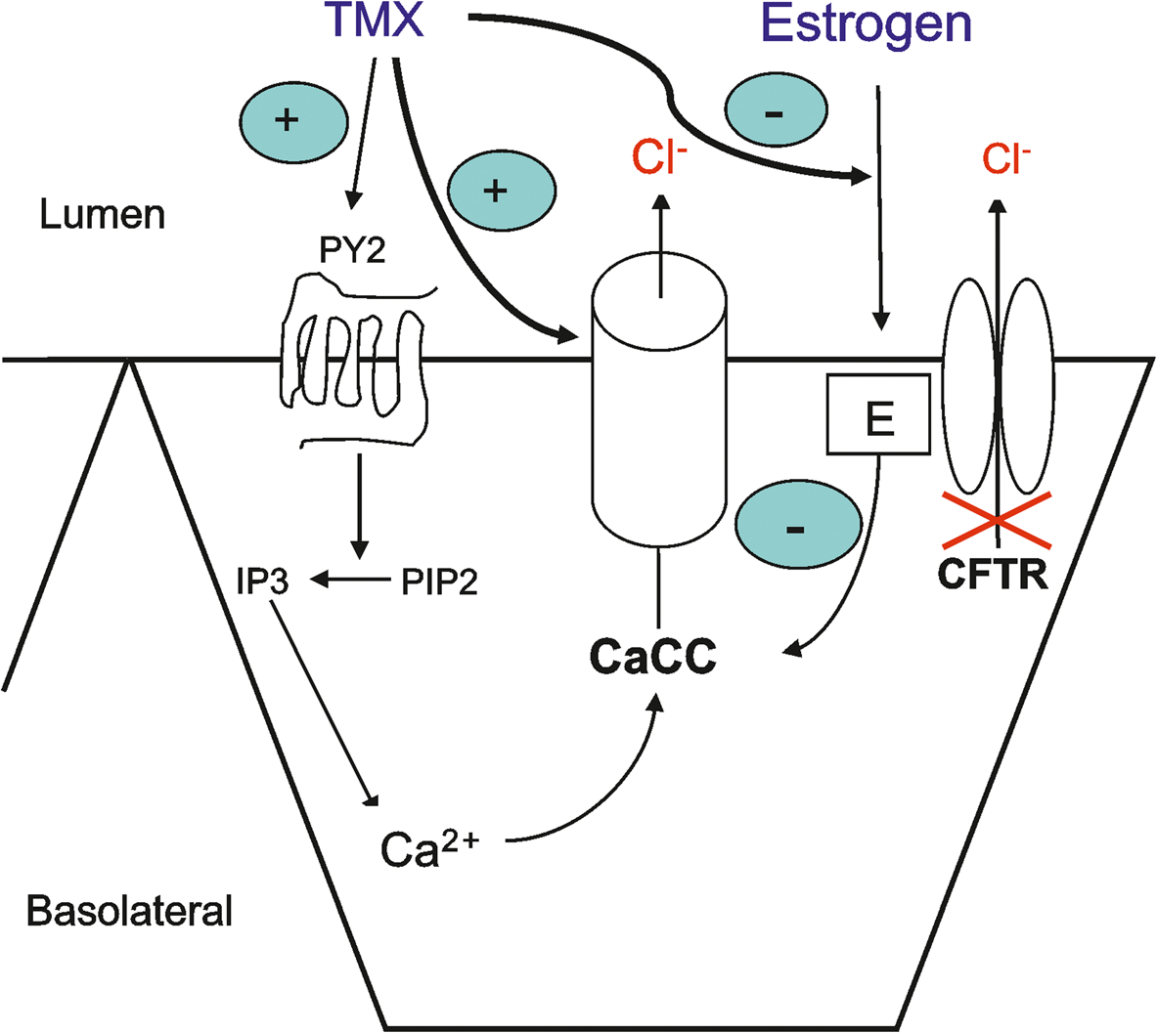
Schematic model of the possible mechanisms of action of tamoxifen. Tamoxifen could be active on different targets. It appears to downregulate estrogen stimulation. On the other hand, tamoxifen interacts with P2Y_2_ purinergic receptors, releasing calcium from the internal stores via IP3 stimulation. However, tamoxifen could upregulate the chloride membrane current by acting directly on CaCC

Our study shows that TMX increases the CaCC currents by two mechanisms: 1) indirectly, as an antagonist of the negative effect of 17β-estradiol on currents, and 2) directly, independently from the antiestrogen effect, acting on the CaCC, the alternate chloride transport pathway (Fig. 9) The effect of TMX on CaCC develops soon after TMX treatment. The CaCC-dependent currents elicited by TMX are quantitatively superior to the CFTR-dependent chloride currents elicited by ivacaftor+lumacaftor. Of note, the combined administration of TMX/ATP and VXs/FSK had an additional effect on chloride currents.

CFTR ion channels in healthy subjects ensure the correct hydration of the respiratory system airway surface. CFTR chloride flux induces an osmotic driven movement of water through the apical membrane of respiratory epithelia. Water release in the lumen dilutes the mucopolysaccharide layer on the luminal cell surface. Fluidity of mucus towards the external environment ensures correct removal of solid residues introduced during breathing [4]. Inflammation and host-defense defect also contribute to the pathophysiology of CF lung disease and lung function deterioration [4].

In CF, CaCC in airway epithelial cells contribute to the secretion of chloride and water. Although the role of CaCC in normal and CF airway epithelial cells has not been completely elucidated, several lines of evidence suggest that CaCC could have clinically relevant effects in CF patients. Mice homozygous for disruption of the *CFTR* genes, unlike their human counterparts, fail to show any gross lung pathology [19]. It is possible that CaCC are more expressed in mice, thus compensating for the lack of CFTR function. By contrast, mice knockout for the *TMEM16A* gene, the gene encoding a CaCC in different secretory epithelia [20–22], exhibit phenotypic abnormalities consistent with a role in fluid and electrolyte homeostasis [23–25]. Finally, in their pivotal study, Knowles and colleagues [26] showed that administration of the CaCC activating agents ATP and UTP in vivo and in vitro was more effective at inducing changes in nasal potential difference in CF patients than in normal controls, suggesting that the activity of CaCC is more pronounced in CF cells than in non-CF cells.

Coakley et al. [10] demonstrated in CF airway epithelial cells that the acute addition of 17ß-estradiol reduced the ATP-induced increase in intracellular Ca^2+^and the production of airway surface liquid. The presence of TMX restored the intracellular Ca^2+^ concentration and potentiated the ATP-induced airway surface liquid secretion, even in the presence of 17ß-estradiol. Our study confirms the results obtained by Coakley et al. [10] and gives new insights on the action of TMX. We explored the possibility of increasing chloride ion fluxes in single epithelial cell cultures, CFBE cells carrying the 508 mutation in homozygosis and IB3–1 cells with the same mutation but in heterozygosis, by stimulating the CaCC. Stimulation of CaCC could be an effective way of creating additional chloride flux in order to promote the movement of water. We found that TMX acts both indirectly, as an antagonist of the negative effect of 17β-estradiol on chloride efflux, and directly, independently of its antiestrogen effect, acting on the CaCC, the alternate chloride transport pathway [17].

TMX, counteracting the effect of 17β-estradiol and acting on the P2Y purinergic receptor, restores the intracellular Ca^2+^ concentration [10]. It is likely that the increase of intracellular Ca^2+^was responsible for the increment of chloride currents observed in our study, which was further amplified by the presence of ATP, a P2Y_2_ activator (Figs. 2, 3, 4, and 5). The whole cell increase of chloride current density was relevant and greater than that elicited by the association of VX-809 + VX-770 plus FSK (Fig. 7). The direct effect of TMX was evident after a few seconds, suggesting that TMX, rather than its metabolites, is responsible for the activation of CaCC. Combined experiments using both VXs/FSK and TMX/ATP (Fig. 8) demonstrated an additional effect on membrane current. This is very important in the prospective of a combined therapeutic use of both treatments. However, the combined action of VXs/FSK and TMX/ATP produced a smaller increment of the membrane permeability than expected from the mathematical addition of the average single current increases due to each compound. There are several possible explanations for this. For example, intracellular calcium mobilization could act on different regulatory pathways. As a consequence, activation of chloride flux by one compound could be responsible for partial activation of other pathways.

To demonstrate that TMX by acting on CaCCs bypasses the CFTR defect, in our study we used two cell lines, CFBE cells, bearing the most frequent mutation F508^−/−^ (class II mutation, defective processing), and IB3–1 cells, bearing the less frequent mutation F508del/W1282X (class I mutation, premature stop codon). We used concentrations of TMX which can be attained in human airways in order to extrapolate our results to the clinical setting. Our findings confirm that TMX bypasses the CFTR defect and, therefore, it might be a curative, mutation-independent treatment applicable to all patients with CF. Adult premenopausal women with CF might gain further advantage from treatment with TMX during the ovulatory period (approximately 1 week per month), when estrogens inhibit CaCC currents [10].

A limit of our study was that it was performed in single cells, rather than in airway epithelial tissue. The effects of TMX on mucociliary transport and inflammation should be studied also in CF animal models. However, “in vitro” cell-based models, which are cost-effective and give preliminary reliable information, have been of immense value for the process of developing new strategies in CF.

## Conclusion

CF is a monogenic disease with a great number of genetic variants of *CFTR*. The effects of environmental factors and modifier genes, which can influence the response to therapeutic agents strongly suggest that the treatment of CF might require different types of drugs in combination. Treatment with TMX might be complementary to and synergistic with other new therapeutic agents such as those acting on the CFTR defect and trafficking, and/or those inhibiting epithelial sodium channel activity. The convergent therapeutic effects of such agents could be highly beneficial for CF patients.

Therapeutic interventions acting on channels alternative to CFTR (e.g., ENaC inhibitors) and a study evaluating the impact of sex hormones (estrogen and progesterone) on lung disease are ongoing [27, 28].

## Abbreviations

17β-estradiol: 1,3,5-estratriene-3,17β-diol or 3,17β-dihydroxy-1,3,5(10)-estratriene
CaCC: Calcium-activated chloride channel
CF: Cystic fibrosis
CFBE: Cell line carrying the F508 point mutation in homozygosis
CFTR: Cystic fibrosis transmembrane conductance regulator
ERα and ERβ: Estrogen receptors
FSK: 7β-acetoxy-8,13-epoxy-1α,6β,9α-trihydroxylabd-14-en-11-one
IB3–1: Cell line carrying the F508del/W1282X mutation
Inh-172: 3-[(3-trifluoromethyl)phenyl]-5-[(4- carboxyphenyl)methylene]-2-thioxo-4-thiazolidinone
NPPB: 5-nitro-2-(3-phenylpropylamino)benzoic acid
TMX: (Z)-1-(p-dimethylaminoethoxyphenyl)-1,2-diphenyl-1-butene
VX-770: Ivacaftor
VX-809: Lumacaftor
ΔPhe508-CFTR: Point mutation of the CFTR protein
